# Human tear proteome dataset in response to daily wear of water gradient contact lens using SWATH-MS approach

**DOI:** 10.1016/j.dib.2021.107120

**Published:** 2021-05-12

**Authors:** Jimmy Ka-Wai Cheung, Jingfang Bian, Ying-Hon Sze, Yee-Kiu So, Wing-Yee Chow, Chun Woo, Ms Tsz-King Wong, King-Kit Li, Thomas Chuen Lam

**Affiliations:** a Centre for Myopia Research, School of Optometry, The Hong Kong Polytechnic University, Hong Kong SAR, China; b Centre for Eye and Vision Research, Hong Kong SAR, China

**Keywords:** Water gradient contact lens, Tears, SWATH, Proteomics

## Abstract

Water Gradient Contact Lens (WGCL) is a new generation material that combines the benefits of Silicone hydrogel (SiHy) and traditional hydrogel contact lenses by modifying the materials between the core and the surface. However, its impact on tear proteome has not been explored. Tears were collected on healthy young adults using Schirmer's strip at baseline, 1-week, and 1-month of WGCL lens wear (*n*=15) and age-matched untouched controls (*n*=10). Equal amounts of tears samples from individuals of WGCL and control groups were randomly pooled to form representative equal parts at each condition (*n*=3 for WGCL wear and age-matched untouched control group) at each condition (baseline, 1-week, and 1-month). Tears were prepared using the S-Trap sample preparation followed by the analysis of a TripleTOF 6600 mass spectrometer. Using Information-dependent acquisition (IDA), a total of 725 tear proteins (6760 distinct peptides) were identified in the constructed spectral library at 1% FDR. Using data-independent acquisition (SWATH-MS), data were analyzed and processed using PeakView (v2.2, SCIEX), with the top differentially expressed proteins at each time point (baseline, 1-week, and 1-month) presented. All acquired raw data (IDA and SWATH-MS) were submitted and published on the Peptide Atlas public repository (http://www.peptideatlas.org/) for general release (Data ID PASS01589).

**Specifications Table**

**Table:** 

Subject	Optometry, Ophthalmology
Specific subject area	Ocular system: Healthy young human subjects with daily wearing water gradient contact lens
Type of data	Table, Graph, Figure
How data were acquired	TripleTOF® 6600 mass spectrometer (SCIEX); IDA and DIA (SWATH-MS) acquisition mode, Raw files searched against UniProt database (Homo Sapiens, organism ID: 9606)
Data format	Raw and Analyzed
Parameters for data collection	Tears were collected from healthy young adults wearing WGCL daily and a cohort of independent, age-matched control at baseline, 1-week, and 1-month.
Description of data collection	Tears were collected from the treatment groups (WGCL lens wear) and age-matched un-touched control at baseline, 1-week, and 1-month. Proteins were prepared using the S-Trap sample preparation. The tryptic peptides were chromatographically separated using the Eksigent ekspert™ nanoLC 415 system and analyzed using the TripleTOF 6600 mass spectrometer (SCIEX). A tear spectral library was generated from the pool samples using information-dependent acquisition (IDA) and quantified using data-independent acquisition (SWATH-MS).
Data source location	Centre for Myopia Research, School of Optometry, The Hong Kong Polytechnic University, Kowloon, Hong Kong
Data accessibility	Repository name: Peptide Atlas public repository Data identification number: PASS01589 Official URL for this dataset: http://www.peptideatlas.org/PASS/PASS01589 Direct URL to data: [ftp://PASS01589: y1017so@ftp.peptideatlas.org/] To access files via FTP, use credentials: Servername: ftp.peptideatlas.org Username: PASS01589 Password: y1017so

## Value of the Data

• This work established an optimized protocol from human tear fluid extraction to SWATH-MS in analyzing contact lens-related tear proteomic changes.
• The generated tear spectral library provided a new open resource for similar anterior eye research using a data-independent acquisition (DIA) approach.
• The tears proteomics dataset with quantitation of temporal protein changes could serve as a novel molecular basis for safety evaluation of the anterior eye condition for future contact lens research.

## 1 Data Description

**Fig. 1:**
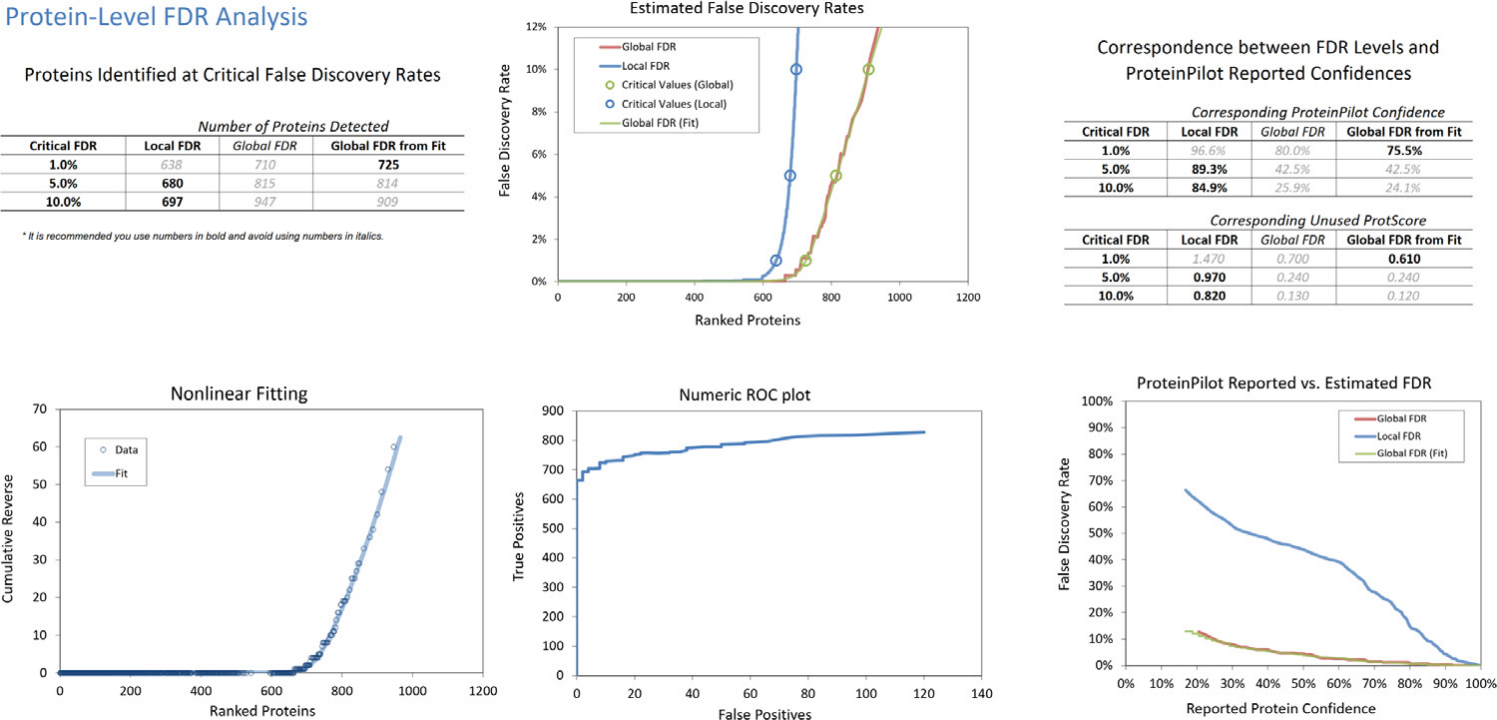
Protein level FDR analysis of human tears (pooled from WGCL wear and age-matched untouched control with 2 repeated injections at each time point, a total of 6 IDA combined searched) proteome identified by ProteinPilot^TM^ software.

**Fig. 2:**
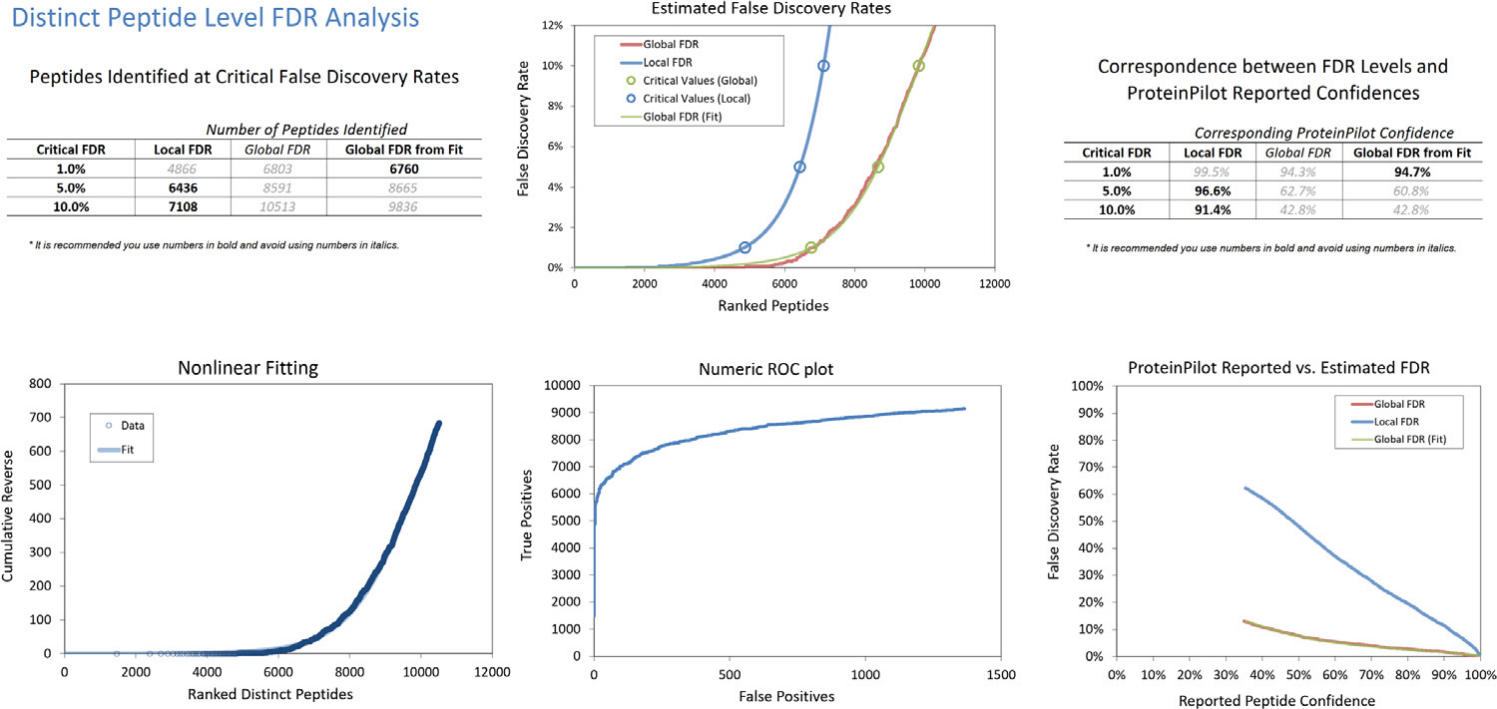
Peptide level FDR analysis of human tears (pooled from WGCL wear and age-matched untouched control with 2 repeated injections at each time point, a total of 6 IDA combined searched) proteome identified by ProteinPilot^TM^ software.

Twenty-five healthy young Chinese adults between age 18 to 22 without known ocular and systematic diseases were recruited and randomly allocated into water gradient contact lens (WGCL) daily wear group and control group without lens wear. Tears proteins at baseline, after 1-week and 1-month WGCL wearing, were screened and analyzed using the SWATH-MS approach. Equal amounts (5 µg) of digested tears samples from individual patients were pooled to form its represented groups (total of *n*=3 for WGCL wear and age-matched untouched control) at each condition (baseline, 1- week, and 1- month) using information- dependant acquisition (IDA) mode, Fig. 1 shows the protein analysis at 1% FDR, and Fig. 2 shows the peptide analysis at 1% FDR. Trypsin digestion enzymatic efficiency is shown in Fig. S1. The full list of identified tears proteins (treatment and control groups with 2 repeated injections at each time point, a total of 6 IDA combined searched) generated from ProteinPilot (V5.0, SCIEX) is shown in Table S1. For SWATH analysis, PeakView (v2.2, SCIEX) was used to process the raw SWATH files and 620 quantified proteins were exported for statistical calculation (Table S2). Table 1 shows the top differentially expressed proteins (Up and Down) at each time point (baseline, 1-week, and 1-month) with the following filter setting: Fold Change (FC) > 1.5 or < 0.7, >1 peptides and P ≤ 0.05 (unpaired T-test). On data repository (http://www.peptideatlas.org/PASS/PASS01589): The DIA_SWATH folder consists of all the individual SWATH injections in raw format (.wiff and .wiff.scan). The IDA folder consists of IDA raw files in different formats (.group, .mgf, .mzid and .xml) and a protein summary report (.xlsx).

**Table 1:** Top differentially expressed proteins (Up-regulated and down-regulated) at each time point (baseline, 1-week, and 1-month) using SWATH-MS in response to WGCL wear to age-matched untouched control with expression ratio, *P*-value.

Time Point	UniProt ID	Gene ID	Protein names	Amino Acid Length	Mass (Da)	FC (CL/Control)	P.value (unpaired equal var)
Baseline	ABC3A_HUMAN	APOBEC3A	DNA dC->dU-editing enzyme APOBEC-3A	199	23,012	2.09	0.007
	AMPN_HUMAN	ANPEP APN, CD13, PEPN	Aminopeptidase N	967	109,540	0.53	0.010

1- week	KV320_HUMAN	IGKV3-20	Immunoglobulin kappa variable 3-20	116	12,557	6.91	0.022
	HORN_HUMAN	HRNR S100A18	Hornerin	2,850	282,390	0.12	0.004

1-month	LV319_HUMAN	IGLV3-19	Immunoglobulin lambda variable 3-19	112	12,042	2.53	0.008
	AMPN_HUMAN	ANPEP APN, CD13, PEPN	Aminopeptidase N	967	109,540	0.51	0.011

## 2 Experimental Design, Materials and Methods

### 2.1 Tears collection

Fifteen subjects prescribed with WGCL as treatments and 10 age-matched control subjects were recruited. Clinical assessments including tears osmolarity, non-invasive keratometric tear film breakup time (NIKTBUT), tear meniscus height (TMH) was recorded at baseline, 1-week, and 1-month visits of WGCL) wear. Tears were collected from subjects at each time point using Schirmer's strips at the tears meniscus close to the inferior temporal eyelids without local anesthesia. Strips were then heated immediately using an optical frame heater for a few minutes until completely dry and stored frozen in 1.5mL polypropylene centrifuge tubes.

### 2.2 Tears protein extraction and preparation

Schirmer's strips were cut into smaller pieces and incubated in 100 μl of 5% SDS at 25°C for 1 hour to elute tears proteins. Rapid gold BCA protein assay (Thermo) was used to determine the protein concentration. Eluted Tears proteins were then prepared and digested using the S-Trap protocol [1], with the use of thermomixer (Eppendorf). In brief, equal amounts (50 μg) of tears proteins were taken from each sample and reduced with dithiothreitol (DTT) to a final concentration of 20 mM at 95°C for 10 mins at 700 rpm using thermomixer (Eppendorf). After cooling to room temperature, samples were then alkylated with iodoacetamide (IAA) to a final concentration of 40mM in the dark at room temperature for 10 mins. Phosphoric acid was then added in a ratio of (1:10, v/v, acid: sample) to a final concentration of 1.2%. Next, S-Trap protein binding buffer (90% MeOH, 0.1M TEAB, pH 7.1) was added to the solution in a 6:1 (v/v, S-Trap: total volume) ratio and mixed thoroughly. The solution was then passed through the S-Trap micro-column and centrifuged at 4000 x g for 30 s. Trapped proteins were then digested with trypsin at a 1:25 ratio (w/w, trypsin: protein) at 47°C for 1 hour. Digested peptides were then eluted in three steps: (A) 40 ul of 50 mM Triethylammonium bicarbonate (TEAB), (B) 40 ul of 0.2% Formic acid (FA) and (C) elution buffer (50% Acetonitrile (ACN), 0.2% FA). The solution was vacuum dried and reconstituted by adding 0.1% FA to equal concentration. Equal amounts (5 µg) of digested tears samples from individual patients were pooled into 3 parts (total of *n*=3 for WGCL wear and age-matched untouched control) at each condition (baseline, 1- week, and 1- month).

### 2.3 LC-MS/MS settings

Both information-dependent acquisition (IDA) and data-independent acquisition (DIA, SWATH-MS) were acquired using the hybrid Quadrupole Time-of-Flight TripleTOF® 6600 mass spectrometer (SCIEX). The MS settings used are similar to our previous studies [2,3]. In brief: Equal amounts of peptides were loaded on to the trap column (100 µm x 2cm, C18) using loading buffer (0.1% Formic acid, 2 % Acetonitrile in water) at 2 µl min-1 for 15 min and on to an analytical column (100 µm x 30 cm, Smartube C18, 5 µm) using an Ekisgent 415 nano-LC system. Gradient running profile was as followed: a flow rate of 350 nL min-1 using mobile phase A (0.1% Formic acid, 2 % Acetonitrile in water) and B (0.1% Formic acid, 98% Acetonitrile in water), 0-0.5 min: 5%B, 0.5-90 min:10%B, 90-120 min:20%B, 120-130 min:28%B, 130-135 min:45%B, 135-141 min:80%B, 141-155 min:5%B. Three microgram peptides were injected into the mass spectrometer with a 10 μm SilicaTip electrospray emitter (New Objective Cat. No. FS360-20-10-N-20-C12). TOF-MS scan range was set at 350 to 1800 m/z with an accumulation time of 250 ms, followed by an MS/MS scan at 100 to 1800 m/z in high sensitivity mode with an accumulation time of 50 ms to up to50 ion candidates per cycle. A threshold of 125 cps was set for MS/MS counting with the charge stage between 2 to 4. For DIA, a setting of 100 variable isolation windows in a looped mode was set over the mass range of 100 to 1800 m/z with an accumulation time of 30 ms, resulting in a total duty cycle <3 sec.

### 2.4 Generation of ion library using information-dependent acquisition (IDA) and SWATH analysis

Two technical replicates of the treatment (WGCL) pool group and the control group from each time points were combined searched (a total of 6 IDA injections) against Homo Sapiens Uniprot database [4] using ProteinPilot (v5.0, SCIEX) with trypsin, iodoacetamide, and biological modification selected in a thorough search effort, along with a 1% false discovery rate (FDR) filter setting. A total of 725 proteins (6760 distinct peptides) were identified at 1% FDR and was loaded as the ion library for SWATH quantification using PeakView (v2.2, SCIEX). Raw SWATH files were loaded onto the ion library via the SWATH Acquisition MicroApp 2.0 and proteins were quantified with the following settings: 6 peptides per protein, 6 transitions per peptide, 90% peptide confidence threshold, 1% FDR, 10 mins XIC extraction window with a width of 75 ppm. MarkerView (v1.3.1, SCIEX) was used to export the Most- likely ratio (MLR) normalized data [5]. For statistical analysis, filters of fold change (FC) > 1.5 fold, >1 peptide and *P* ≤ 0.05 (unpaired T-test) were applied for the proteins to be classified as significantly different.

## Ethics Statement

All procedures for the sample collection of tears and handling were obtained from patients and approved by the Human Subjects Ethics Sub-Committee of The Hong Kong Polytechnic University [#HSEARS20190416033].

## CRediT Author Statement

**Jimmy Ka-Wai Cheung, Jingfang Bian and Ying-Hon Sze:** Formal analysis, Validation, Writing – original draft; **Yee-Kiu So, Wing-Yee Chow and Chun Woo:** Investigation, Methodology, Data curtion; **Ms Tsz-King Wong:** Supervision; **King-Kit Li:** Validation; **Thomas Chuen Lam:** Conceptualization, Methodology, Resources, Writing – review & editing, Funding acquisition.

## Declaration of Competing Interest

The authors declare that they have no known competing financial interests or personal relationships that could have appeared to influence the work reported in this paper.

## Appendix Supplementary materials

Image, application 1

Image, application 2

Image, application 3
