# Supplementary figures and images for: Human tear proteome dataset in response to daily wear of water gradient contact lens using SWATH-MS approach
Source: Data Brief. 2021 May 12;36:107120. doi: 10.1016/j.dib.2021.107120 (PMC8165404; doi:10.1016/j.dib.2021.107120)

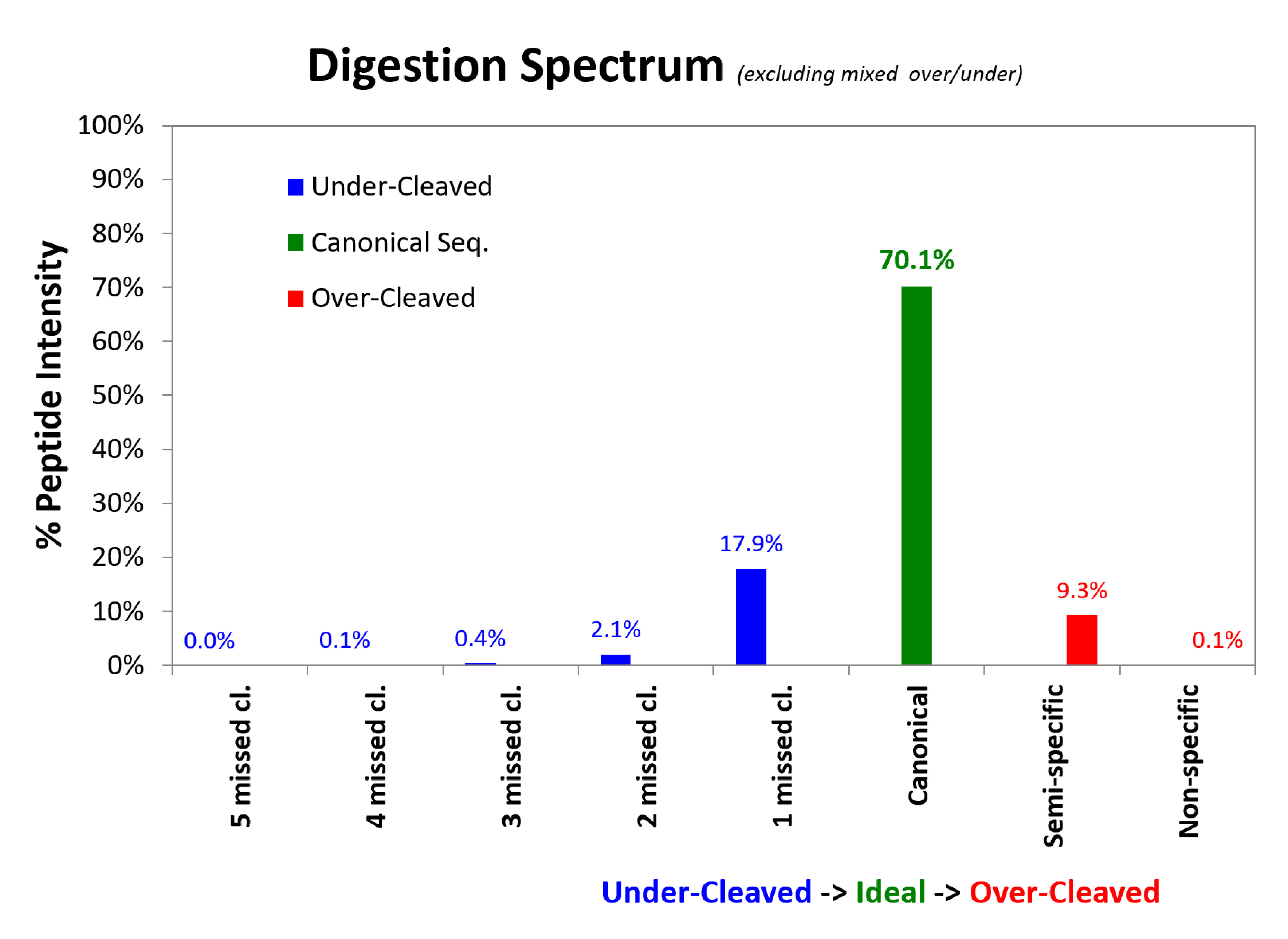

Supplement: Supplementary file 3 [file mmc3.jpg]
